# Combination of Fibrosis-4, liver-stiffness measurement, and Fibroscan-AST score to predict liver-related outcomes in nonalcoholic fatty liver disease

**DOI:** 10.1097/HC9.0000000000000244

**Published:** 2023-09-22

**Authors:** Yu Jun Wong, Esteban Urias, Michael W. Song, Tanvi Goyal, Wei Xuan Tay, Nicole Xinrong Han, Jing Hong Loo, Tian Yu Qiu, Karn Wijarnpreecha, Yiong Huak Chan, Vincent L. Chen

**Affiliations:** 1 Department of Gastroenterology & Hepatology, Changi General Hospital, Singapore; 2 Duke-NUS Academic Clinical Program, SingHealth, Singapore; 3 Yong Loo Lin School of Medicine, National University of Singapore, Singapore; 4 Division of Gastroenterology and Hepatology, University of Michigan, Ann Arbor, Michigan, USA; 5 Department of Medicine, Division of Gastroenterology and Hepatology, University of Arizona College of Medicine, Phoenix, Arizona, USA; 6 Biostatistics Unit, Yong Loo Lin School of Medicine, National University of Singapore, Singapore

## Abstract

**Introduction:**

Noninvasive tests, such as Fibrosis-4 (FIB-4), liver-stiffness measurement (LSM) by vibration-controlled transient elastography, and Fibroscan-AST (FAST), are frequently used for risk stratification in NAFLD. The comparative performance of FIB-4 and LSM and FAST to predict clinical outcomes of patients with NAFLD remained unclear. We aim to evaluate the performance of FIB-4, LSM, and FAST scores to predict clinical outcomes in patients with NAFLD.

**Methods:**

We included consecutive adult patients with NAFLD with transient elastography performed between 2015 and 2022 from the United States and Singapore. Patients with NAFLD stratified based on baseline FIB-4, LSM, and FAST score were followed up until clinical outcomes notably liver-related events (LREs), LREs or death, death, and major adverse cardiac events.

**Results:**

A total of 1262 patients with NAFLD (63% with obesity and 37% with diabetes) with vibration-controlled transient elastography were followed up for median 3.5 years. FIB-4 stratified patients with NAFLD into low-risk (<1.3), intermediate-risk (1.3–2.67), and high-risk (>2.67) in 59.4%, 31.5%, and 9.1%, respectively. No LRE occurred with baseline FIB-4 <1.3, regardless of LSM and FAST score. Higher FIB-4 was associated with a higher risk of LREs within each LSM category. FIB-4 had a higher area under the received operating characteristic curve than LSM or FAST score to predict LRE.

**Conclusions:**

In this multicenter international study, FIB-4 and LSM synergistically predicted the risk of LRE. In patients with FIB-4 <1.3, vibration-controlled transient elastography may incorrectly classify up to 10% of the patients as high risk. FIB-4 should be incorporated into risk stratification in NAFLD even among patients who underwent VCTE.

## INTRODUCTION

NAFLD affects nearly one-third of the global population^1^ and is a leading indication for liver transplantation.^2^ However, most patients with NAFLD do not develop decompensated liver disease, highlighting the importance of cost-effective risk stratification strategies so we can effectively identify high-risk patients with NAFLD without overwhelming tertiary care centers with low-risk patients.^3,4^

Current guidelines recommend the sequential use of the Fibrosis-4 (FIB-4) score followed by liver-stiffness measurement (LSM) by vibration-controlled transient elastography (VCTE) to risk-stratify patients with NAFLD.^5^ While sequential testing using noninvasive tests (NITs) has been shown to improve the classification of patients with NAFLD into fibrosis stages,^6^ NITs frequently yield discordant results and it remains unclear how to interpret such discrepancies. It also remains unclear if the combination of LSM and FIB-4 or LSM alone improves on the performance of FIB-4 alone to predict clinical outcomes in patients with NAFLD. While LSM-based strategies correlate with clinical outcomes among viral-associated and alcohol-associated patients with cirrhosis,^7^ such data remain limited among patients with NAFLD.^8^ There are also concerns of the lower accuracy of LSM among obese or low-risk patients with NAFLD.^9,10^

In addition to risk stratification based on the fibrosis stage, accurately identifying patients with high-risk NASH is important to identify potential clinical trial participants. Traditionally, the diagnosis of NASH requires a liver biopsy.^3^ More recently, combinations of NITs have been proposed to identify at-risk patients with NASH beyond stage 2 fibrosis, such as the Fibroscan-AST (FAST) score,^11^ even though its external validation remained limited. The prognostic value of FAST score is of great interest because it provided a noninvasive alternative to liver biopsy to evaluate both the inflammatory and fibrosis burden in patients with NAFLD. Moreover, it is not known whether the FAST score predicts clinical events in patients with NAFLD, especially when used in conjunction with FIB-4 or LSM.

Therefore, in this study, we aim to determine the prognostic significance of the FIB-4, LSM, and FAST scores to predict liver-related events (LRE: defined as hepatic decompensation, HCC, or liver transplantation) among patients with NAFLD from Asian and western centers. We also aim to perform sensitivity analysis to determine the prognostic significance of these NITs in predicting death, LRE/death, and major adverse cardiac events (MACEs).

## METHODS

### Study population

This is a multicenter, retrospective cohort study of consecutive adults (age above 18 y) with NAFLD from the University of Michigan Health System (United States of America) and Changi General Hospital (Singapore) who underwent VCTE between January 1, 2015, and December 31, 2022. The study was approved by the respective institutional ethics committees with waiver of consent granted, and was conducted in compliance with the Declarations of Helsinki and Istanbul.

NAFLD was diagnosed based on either radiological (ultrasound, CT or MRI) or histological diagnosis of hepatic steatosis, without documented alternative chronic liver disease or significant alcohol intake (defined as >1 U/d in female or >2 U/d in male). Clinical data were collected using a unified data template.

### Noninvasive assessments (FIB-4, VCTE, and FAST)

FIB-4 was calculated using patients age at the time of LSM and baseline laboratory results within 6 months of LSM.^12^ LSMs were performed using VCTE by certified operators using either a M or XL probe, based on the manufacturer’s instruction. LSM was measured as the median of at least 10 successful measurements, expressed in kilopascals (kPa). We defined unreliable LSMs as interquartile range >30% of the median LSM value or <10 successful measurements. The FAST score was computed using a combination of serum AST, ALT, LSM and controlled attenuation parameter score measured by FAST.^11^ The predictors were FIB-4 score, stratified by low FIB-4 (<1.3), intermediate FIB-4 (1.3–2.67), and high FIB-4 (>2.67);^13^ FAST, stratified by <0.35, 0.35–0.67, and >0.67; and LSM, stratified by low LSM (<8 kPa), intermediate LSM (8–12 kPa), and high LSM (>12 kPa).^7^ All clinical events were manually reviewed to ensure data accuracy.

### Outcomes measures

Our primary outcome was the occurrence of the first LREs, which were defined as the occurrence of liver decompensation (variceal bleeding, clinically overt ascites or overt HE), HCC, or liver transplantation.^13^ Variceal bleeding was confirmed from endoscopy and consultation reports. Ascites was defined as clinically overt ascites requiring diuretic treatment, large-volume paracentesis, or transjugular intrahepatic shunt placement. Overt HE was defined by West Haven Classification grade 2 and beyond by the managing specialists. We included three secondary outcomes. First, given that most patients with NAFLD do not die of liver disease,^14^ we included a composite end point of either LRE or all-cause mortality. Second, we included an outcome of all-cause mortality. Third, MACE were defined as a composite end point of myocardial infarction, coronary revascularization, heart failure requiring hospitalization or stroke.^15^ All clinical events were manually reviewed for verification. We excluded patients with less than 6 months of follow-up or events that occurred within the first 6 months of the study to avoid misclassifying prevalent disease as incident.

### Statistical analysis

We summarized the baseline characteristics of our cohort based on study sites. Continuous data were reported in mean ± SD or median with interquartile range based on normality of data distribution. Categorical data were summarized by frequency (percentage). Numerical and categorical baseline variables comparisons using 2 Sample T or Mann-Whitney *U* tests and the Chi-square/Fisher exact tests, respectively.

Kaplan-Meier with log-rank test was used to compare the time to event variables across groups. We reported both the cumulative incidence and the incidence rate (reported as events in 1000 per person-year) with the respective 95% CI between different subgroups (FIB-4, LSM, or FAST). The diagnostic statistics of NITs and combinations of NITs to predict clinical outcomes were reported. The time-dependent area under the received operating characteristic curve (tAUC) at 3 years between different NITs was compared using Delong test at various optimal cutoff using (1) Youden Index, (2) sensitivity ≥90%, and (3) specificity ≥90%.^16,17^ Sensitivity analysis was performed to compare the tAUC of NITs to predict LRE or LRE/death at 5 years. We estimated the risk of developing LRE using the Fine-Gray competing risk regression, with death as competing risk, and expressed in subdistributional HR (with 95% CI.^18^ To determine the performance of NITs in identifying low-risk patients with NAFLD, we compared the misclassification of low-risk NAFLD between NITs by performing the test of marginal homogeneity. Statistical analysis was performed using STATA/SE version 17.0 (StataCorp LLC, USA) and R version 4.1.2 (R Foundation for Statistical Computing).

## RESULTS

### Baseline demographics

A total of 1262 patients with NAFLD were included from the United States and Singapore (Supplemental Figure S1, http://links.lww.com/HC9/A486). The cohort was predominantly White (62%) with a mean age of 52 years (Table 1). The mean (±SD) body mass index was 31.9 (±6.7) kg/m^2^, with 63.1% of the population having obesity and one-third having diabetes mellitus. The median (interquartile range) follow-up was 3.5 (2.4–4.6) years with 4342 person-years of follow-up in total. Despite a lower proportion of patients with NAFLD with obesity (67.9% vs. 46.0%, *p* < 0.0001) when compared to the US cohort, the Singapore NAFLD cohort was older with more metabolic comorbidities such as diabetes mellitus, hypertension, and hyperlipidemia (*p* < 0.0001 for all) (Table 1). The Singapore NAFLD cohort also had a lower baseline ALT (52U/L vs. 73U/L, *p* = 0.004), lower AST (39 U/L vs. 48 U/L, *p* = 0.004), but a higher serum creatinine (82 mmol/L vs. 78 mmol/L, *p* = 0.019) than the US cohort.

**TABLE 1:** Baseline characteristics of NAFLD patients from the Michigan and Singapore cohorts

	Total cohort	US cohort	Singapore cohort	
Variable	(N = 1262)	(N = 990)	(N = 272)	*p*
Demographics				
Age^a^	51.8 ± 13.9	50.5 ± 13.6	56.7 ± 14.1	<0.001
Male^b^, n (%)	668 (52.9)	516 (52.1)	152 (55.9)	0.271
Race^b^, n (%)				
Asian	323 (25.6)	72 (7.3)	251 (92.3)	<0.001
Black	39 (3.1)	39 (3.9)	0	—
Hispanic	62 (4.9)	62 (6.3)	0	—
White	787 (62.4)	786 (79.4)	1 (0.4)	—
Others	51 (4.0)	31 (3.1)	20 (7.4)	—
Comorbidities^b^, n (%)				
Diabetes mellitus	469 (37.2)	333 (33.6)	136 (50.0)	<0.001
Hypertension	662 (52.5)	498 (50.3)	164 (60.3)	0.003
Hyperlipidemia	728 (57.7)	520 (52.5)	208 (76.5)	<0.001
BMI^b^, n (%)	—	—	—	<0.001
Lean	103 (8.5)	59 (6.2)	44 (16.2)	—
Overweight	347 (28.5)	244 (25.8)	103 (37.9)	—
Obese, class I	382 (31.4)	288 (30.5)	94 (34.6)	—
Obese, class II	227 (18.7)	210 (22.2)	17 (6.3)	—
Obese, class III	158 (13.0)	144 (15.2)	14 (5.1)	—
Laboratory values^a^				
ALT (U/L)^a^	68.2 ± 103.5	72.7 ± 115.0	52.0 ± 35.6	0.004
AST (U/L)^a^	46.1 ± 46.1	48.1 ± 50.5	39.0 ± 22.6	0.004
Albumin (mg/dL)^a^	44.5 ± 3.9	44.4 ± 3.6	44.9 ± 4.8	0.075
Total bilirubin (mg/dL)^a^	12.3 ± 13.9	12.6 ± 15.3	11.3 ± 6.7	0.189
Creatinine (mmol/L)^a^	78.6 ± 26.1	77.7 ± 19.4	82.2 ± 43.8	0.017
Platelet count (10^3^/μL)^a^	241.5 ± 73.3	241.1 ± 73.3	242.8 ± 81.8	0.732
FIB-4^a^	1.42 ± 1.07	1.38 ± 1.00	1.57 ± 12.8	0.010 0.011
FIB-4^b^				
<1.3	749 (59.4)	608 (61.4)	141 (51.8)	—
1.3–2.67	398 (31.5)	300 (30.3)	98 (36.0)	—
>2.67	115 (9.1)	82 (8.3)	33 (12.1)	0.280
LSM (kPa)^a^	9.0 ± 7.6	9.1 ± 7.8	9.0 ± 6.5	0.901
<8	771 (61.1)	661 (61.7)	160 (58.8)	0.194
8–12	264 (20.9)	221 (21.3)	53 (19.5)	—
>12	227 (18.0)	168 (17.0)	59 (21.7)	—
FAST score	0.43 ± 0.24	0.44 ± 0.23	0.37 ± 0.26	<0.001
FAST	536 (42.5)	395 (39.9)	141 (51.8)	0.001
<0.35	479 (38.0)	398 (40.2)	81 (29.8)	—
0.35–0.67	247 (19.5)	197 (19.9)	50 (18.4)	—
>0.67	—	—	—	—
Follow-up duration (y)	3.4 ± 1.5	3.4 ± 1.5	3.8 ± 1.6	<0.001

a Mean (SD) or median (IQR).

b Number (%).

Abbreviations: ALT, alanine aminotransferase; AST, aspartate aminotransferase; BMI, body mass index; FAST, Fibroscan-AST; FIB-4, Fibrosis index 4; LSM, liver-stiffness measurement.

The LSM met quality criteria in 99.6% of the patients and 48.2% required XL probe. The mean (±SD) LSM was 9.0 kPa (±7.6 kPa), which was comparable between the US and Singapore cohort. The LSM stratified patients with NAFLD into 3 subgroups of low (<8 kPa), intermediate (8–12 kPa), and high LSM (>12 kPa) in 61.1%, 20.9%, and 18.0%, respectively. The FIB-4 score stratified patients with NAFLD into 3 subgroups of low-risk (<1.3), intermediate-risk (1.3–2.67), and high-risk (>2.67) in 59.4%, 31.5%, and 9.1%, respectively. The Singapore cohort had a higher mean FIB-4 (1.57 vs. 1.38, *p* = 0.010) and lower mean FAST score (0.37 vs. 0.44, *p* < 0.001) than the US cohort, which is likely driven by the difference in age and baseline ALT between the 2 groups.

### Incidence of clinical outcomes based on NITs

#### Liver-related events

**FIGURE 1:**
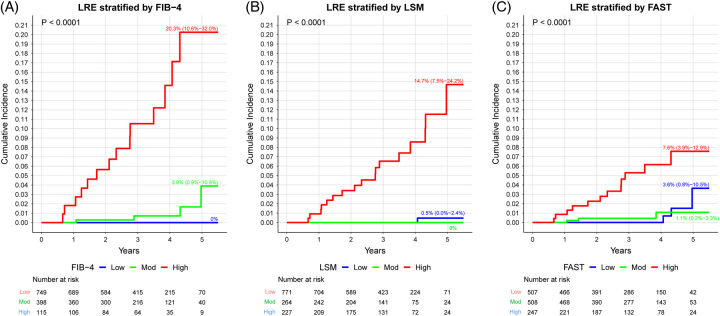
Cumulative incidence of liver-related events based on FIB-4, LSM, or FAST score. The 5-year cumulative incidence of liver-related event was higher among NAFLD patients with a high FIB-4 score, high LSM, or high FAST score. FIB-4, low (<1.3), intermediate (1.3–2.6), high (>2.6); LSM: low (<8 kPa), intermediate (8–12 kPa), high (>8 kPa); FAST: low (<0.35), intermediate: (0.35–0.67), high (>0.67). Abbreviations: FAST, Fibroscan-AST score; FIB-4, Fibrosis index of 4 factors; LRE, liver-related events; LSM, liver-stiffness measurement.

The overall incidence of LRE was 4.66 (95% CI, 3.00–7.23) per 1000 person-years. The number of patients developing each LRE and liver-related death were as follows: ascites (n = 21), variceal bleeding (n = 9), HE (n = 10), HCC (n = 10), liver transplantation (n = 0), and liver-related death (n = 3) (Supplemental Table S1, http://links.lww.com/HC9/A486). Overall, there was a steady increase in the risks of LREs with increasing FIB-4, LSM, and FAST scores (Figure 1). The incidence rate (per 1000 person-year) of LRE in patients with NAFLD with low-risk FIB-4 (<1.3), low LSM (<8 kPa), and low FAST score (<0.35) was 0.0 (95% CI, 0–1.6) per 1000 person-years, 0.4 (95% CI, 0–2.3) per 1000 person-years, and 1.9 (95% CI, 0.4–5.5) per 1000 person-years, respectively (Table 2). Increasing FIB-4 was associated with a significantly higher incidence rate of LRE per 1000 person-year: 0.0 (95% CI, 0.0–1.6), 3.2 (95% CI, 0.9–8.3), and 39.5 (95% CI, 21.6–66.3) for low, intermediate, and high-risk FIB-4, respectively (*p* < 0.001). The incidence rate of LRE per 1000 person-year also increases with LSM: 0.4 (95% CI, 0.1–2.3), 0 (95% CI, 0–4.5), and 23.3 (95% CI, 13.6–37.3) for low, intermediate, and high-risk FIB-4, respectively (*p* < 0.001).

**TABLE 2:** The 5-year cumulative incidence of liver-related events and death stratified based on FIB-4, FAST score, and liver-stiffness measurement

	FIB-4								
	<1.3		1.3–2.67		>2.67			All patients	
Liver-related events	5-y cumulative incidence (n = 749) (%)	Incidence rate per 1000 person-years	5-y cumulative incidence (n – 398) (%)	Incidence rate per 1000 person-years	5-y cumulative incidence (n = 115) (%)	Incidence rate per 1000 person-years	*p* ^a^	5-y cumulative incidence (%)	Incidence rate per 1000 person-years
LSM (kPa)									
<8	0	0 (0.0–0.2)	0	0.00 (0.00–5.89)	14.3 (0.5–49.1)	14.03 (0.36–78.17)	<0.001	0.5 (0.0–2.4)	0.42 (0.01–2.33)
8–12	0	0(0.0–0.8)	0	0.00 (0.00–11.57)	0	0.00 (0.00–45.45)	NA	0	0.00 (0.00–4.53)
>12	0	0 (0.0–1.4)	15.6 (3.0–37.5)	13.78 (3.75–35.28)	29.2 (15.2–44.6)	64.48 (34.33–110.26)	< 0.001	14.7 (7.5–24.2)	23.30 (13.57–37.31)
*p*	NA		0.0017		0.019		—	<0.001	
All patients	0	0.00 (0.00–1.57)	3.9 (0.9–10.9)	3.24 (0.88–8.29)	20.3 (10.6–32.0)	39.54 (21.62–66.34)	<0.001	3.3 (1.7–5.8)	4.58 (2.71–7.24)
FAST score									
<0.35	0	0 (0.0–0.3)	13.8 (1.3–40.4)	5.33 (0.65–19.26)	14.3 (0.5–49.1)	21.14 (0.54–117.81)	<0.001	3.6 (0.8–10.5)	1.89 (0.39–5.53)
0.35–0.67	0	0 (0.0–0.4)	0	0.00 (0.00–7.00)	19.6 (2.4–49.1)	31.59 (6.51–92.32)	< 0.001	1.1 (0.2–3.3)	1.91 (0.39–5.58)
>0.67	0	0 (0.0–1.5)	2.6 (0.5–8.4)	6.00 (0.73–21.68)	21.2 (9.9–35.4)	47.21 (22.64–86.83)	<0.001	7.6 (3.9–12.9)	15.50 (8.01–27.08)
*p*	NA		0.20		0.65			< 0.001	
All patients	0	0.00 (0.00–1.57)	3.9 (0.9–10.9)	3.24 (0.88–8.29)	20.3 (10.6–32.0)	39.54 (21.62–66.34)	<0.001	3.3 (1.7%–5.8%)	4.58 (2.71–7.24)
Liver-related events or death									
LSM (kPa)									
** **<8	0	0 (0.0–0.2)	1.4 (0.3–4.5)	3.20 (0.39–11.54)	14.3 (0.5–49.1)	14.03 (0.36–78.17)	0.0016	0.8 (0.2–2.5)	1.26 (0.26–3.67)
** **8–12	0	0 (0.0–0.8)	0	0.00 (0.00–11.57)	8.0 (1.3–22.9%)	24.64 (2.98–89.01)	<0.001	0.8 (0.2–2.7%)	2.46 (0.30–8.87)
** **>12	0	0 (0.0–0.01)	15.6 (3.0–37.5)	13.78 (3.75–35.28)	29.2 (15.2–44.6)	64.48 (34.33–110.26)	<0.001	14.7 (7.5–24.2)	23.30 (13.57–37.31)
** ** *p*^b^	—	—	0.037		0.14		—	<0.001	<0.001
** **All patients	0	0 (0.00–1.57)	4.6 (1.3–11.3)	4.86 (1.78–10.57)	22.1 (12.1–33.8)	45.19 (25.83–73.38)	<0.001	3.7 (2.0–6.1)	5.60 (3.51–8.47)
** **FAST									
** **<0.35	0	0 (0.0–0.3)	16.0 (2.2– 1.5)	10.66 (2.90–27.30)	14.3 (0.5–49.1)	21.14 (0.54–117.81)	0.001	4.2 (1.1–10.8)	3.15 (1.02–7.36)
** **0.35–0.67	0	0 (0.0–0.4)	0	0 (0.00–7.00)	22.6 (3.7–51.3)	42.12 (11.48–107.84)	< 0.001	1.3 (0.4–3.5)	2.55 (0.69–6.52)
** **>0.67	0	0 (0.0–1.4)	2.6 (0.5–8.4)	6.00 (0.73–21.68)	22.8 (11.1–37.0)	51.94 (25.93–92.93)	< 0.001	8.0 (4.2–13.4)	16.80 (8.94–28.72)
** ** *p*	NA		0.056		0.62		—	<0.001	
** **All patients	0	0 (0.0–0.1)	4.6 (1.3–11.3)	4.86 (1.78–10.57)	22.1 (12.1–33.8)	45.19 (25.83–73.38)	< 0.001	0.7 (2.0–6.1)	5.60 (3.51–8.47)

*Note:* Incidence rate is shown as events in 1000 per person-years (95% CI) in the overall cohort. Person-years was rounded to the nearest.

a *p*-value reflects differences between cumulative incidence between different subgroups of FIB-4, LSM, and/or FAST using Fisher-exact test. 5-year cumulative incidence is shown as events/number at risk (%, 95% CI).

Abbreviations: FAST, Fibroscan-AST; FIB-4, Fibrosis index 4; LRE, liver-related events; LSM, liver-stiffness measurement.

Subgroup analysis showed that higher FIB-4 score was associated with a higher incidence rate (per 1000 person-years) of LRE in those with high LSM: 0.0 (95% CI, 0.0–1.4), 13.8 (95% CI, 3.8–35.3), and 64.5 (95% CI, 34.3–110.3) for low, intermediate, and high risk, respectively. In those with high FAST score, a higher FIB-4 and LSM were associated with a higher incidence rate of LRE. The incidence rate of LRE (per 1000 person-years) stratified by FIB-4 was 0.0 (95% CI, 0.0–1.5), 6.0 (95% CI, 0.7–21.7), and 47.2 (95% CI, 22.6–86.6) for low, intermediate, and high FIB-4, respectively (*p* < 0.001).

In a multivariable regression model, the independent predictors of developing LRE include FIB-4 > 2.67 (HR: 9.8, 95% CI, 3.5–27.3), hypertension (HR: 4.1, 95% CI, 1.1–15.3), and LSM > 8 kPa (HR: 4.5, 95% CI, 1.2–17.6) (Supplemental Table S2, http://links.lww.com/HC9/A486).

#### LREs or death

The overall incidence rate of LREs/death was 6.30 (95% CI, 4.32–9.18) per 1000 person-years. The incidence rate in patients with FIB-4 < 1.3, LSM < 8 kPa and FAST score < 0.35 was 0.0 (95% CI, 0–1.6) per 1000 person-years, 1.3 (95% CI, 0.3–3.7) per 1000 person-years, and 3.2 (95% CI, 1.0–7.4) per 1000 person-years, respectively. Overall, there was a steady increase in the risk of LRE/death with increasing FIB-4, LSM, and FAST score (Table 2, Supplemental Figure S2, http://links.lww.com/HC9/A486). Of note, there were no LREs or deaths in patients with NAFLD with low-risk FIB-4 regardless of the LSM or FAST value, after 2566 person-years of follow-up (Table 2).

Subgroup analysis showed that FIB-4 stratified the risk of LRE/death among “high-risk” patients with NAFLD (Table 2). Among those with LSM> 12 kPa, the incidence rate of LRE/death (per 1000 person-years) was 0.0 (95% CI, 0.0–0.01), 13.8 (95% CI, 3.8–35.3), and 64.5 (95% CI, 34.3–110.3) for low, intermediate, and high FIB-4, respectively (*p* < 0.001). Among those with FAST score > 0.67, the incidence rate of LRE/death (per 1000 person-years) was 0.0 (95% CI, 0.0–1.4), 6.0 (95% CI, 0.7–21.7), and 52.0 (95% CI, 25.9–92.9) for low, intermediate, and high FIB-4, respectively (*p* < 0.001).

#### Death and MACE

The overall cumulative incidences of death and MACE were 1.84 (95% CI, 0.92–3.68) and 2.33 (95% CI, 1.25–4.33) per 1000 person-years, respectively. The causes of death are summarized in Supplemental Table S3, http://links.lww.com/HC9/A486. Neither FIB-4, LSM, nor FAST was associated with the incidence of death or MACE (Table 2, Supplemental Table S4, http://links.lww.com/HC9/A486).

#### Diagnostic accuracy of FIB-4, LSM, and FAST

**FIGURE 2:**
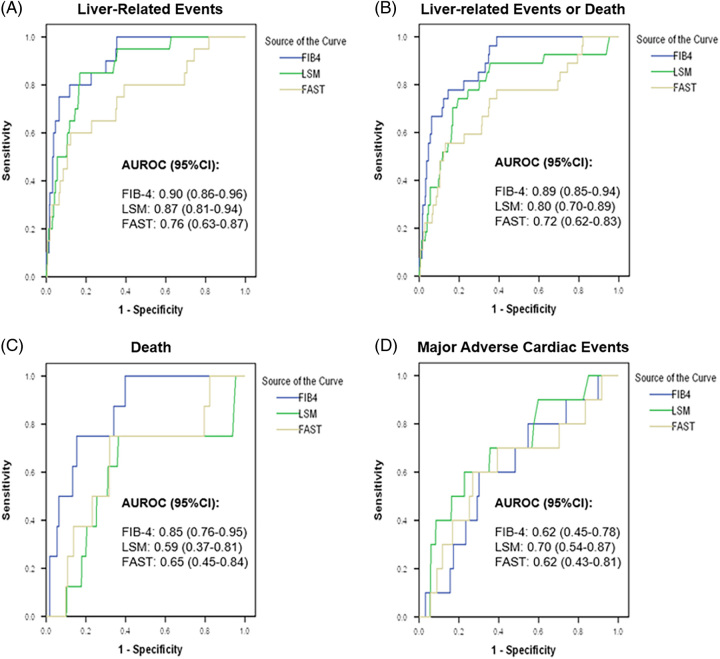
Time-dependent ROC curves for prediction of liver-related events, liver-related events/death, death, and major adverse cardiac events at 3 years using FIB-4, LSM, and FAST. Abbreviations: FAST, Fibroscan-AST score; FIB-4, Fibrosis index of 4 factors; LRE, liver-related events; LSM, liver-stiffness measurement.

The diagnostic accuracy of FIB-4, LSM, and FAST score in predicting various clinical outcomes is summarized in Table 3. While both FIB-4, LSM, and FAST could stratify the 3-year cumulative incidence of LRE in NAFLD, FIB-4 has higher accuracy than FAST score in predicting LRE, LRE/death, and death at 3 years when the tAUC was determined at the optimal cutoff based on the Youden index (Table 3, Figure 2), sensitivity ≥90% (Supplemental Table S5, http://links.lww.com/HC9/A486), or specificity ≥90% (Supplemental Table S6, http://links.lww.com/HC9/A486). While the predictive accuracy for LREs was comparable between FIB-4 and LSM, FIB-4 has a higher tAUC than FAST score to predict LRE at 3 years and 5 years (3 y: FIB-4: 0.90 vs. 0.76, *p* = 0.006; 5 y: FIB-4: 0.94 vs. 0.83, *p* = 0.034). Moreover, FIB-4 is also significantly more accurate to predict LRE/death at 3 years and 5 years than both LSM and FAST score (Supplemental Table 7, http://links.lww.com/HC9/A486). FIB-4 also had higher tAUC for death than LSM (FIB-4: 0.85 vs. 0.59, *p* = 0.068) and FAST (FIB-4: 0.85 vs. 0.65, *p* = 0.038) at 3 years (Figure 2). All 3 scores had limited ability to predict MACE (tAUC 0.62–0.70).

**TABLE 3:** Accuracy of FIB-4, LSM, and FAST score to predict clinical outcomes at 3 years

	tAUC^a^ (95% CI)	Youden Index	Sensitivity, % (95% CI)	Specificity, % (95% CI)	PPV, % (95% CI)	NPV, % (95% CI)	*p* of AUC vs. FIB-4
Liver-related events							
FIB-4	0.939 (0.881–0.993)	2.1	91.7 (61.5–99.8)	85.5 (83.4–87.4)	5.7 (2.9–10.0)	99.9 (99.5–100)	Reference
LSM	0.876 (0.812–0.956)	11.4	83.3 (51.6–97.9)	78.5 (76.1–80.7)	3.6 (1.7–6.5)	99.8 (99.3–100)	0.143
FAST score	0.842 (0.702–0.958)	0.73	66.7 (34.9–90.1)	87.1 (85.1–88.9)	4.7 (2.1–9.1)	99.6 (99.1–99.9)	0.034
Liver-related events or death							
FIB-4	0.903 (0.845–0.958)	1.9	84.2 (60.4–96.6)	82.8 (80.6–84.8)	7.0 (4.0–11.0)	99.7 (99.1–99.9)	Reference
LSM	0.747 (64.8–89.9)	8.3	89.5 (66.9–98.7)	64.1 (61.4–66.8)	3.7 (2.1–5.8)	99.8 (99.1–199)	0.024
FAST score	0.751 (0.634–0.880)	0.53	73.3 (48.8–90.8)	66.0 (63.3–68.6)	3.2 (1.8–5.3)	99.4 (98.6–99.8)	0.006
Death							
FIB-4	0.855 (0.750–0.954)	1.9	75.0 (34.9–96.8)	82.1 (79.9–84.2)	2.6 (1.0–5.6)	99.8 (99.3–100)	Reference
LSM	0.549 (0.352–0.822)	7.9	75.0 (34.9–96.8)	61.0 (58.2–63.7)	1.2 (0.4–2.6)	99.7 (99.1–100)	0.021
FAST score	0.612 (0.441–0.850)	0.53	75.0 (34.9–96.8)	65.6 (62.9–68.2)	1.4 (0.5–3.0)	99.8 (99.1–100)	0.038
Major adverse cardiac events							
FIB-4	0.654 (0.454–0.842)	1.5	62.5 (24.5–91.5)	68.5 (65.8–71.1)	1.3 (0.4–2.9)	99.7 (99.0–99.9)	Reference
LSM	0.670 (0.468–0.884)	10.0	62.5 (24.5–91.5)	74.5 (72.1–77.0)	1.5 (1.3–9.2)	99.7 (99.1–99.9)	0.703
FAST score	0.573 (0.334–0.819)	0.57	50.0 (15.7–84.3)	70.6 (68.0–73.0)	1.1 (0.3–2.7)	99.6 (98.8–99.9)	0.513

*Note:* tAUC was compared using the Delong test.

a tAUC, time-dependent area under the received operating curve.

Abbreviations: FAST, Fibroscan-AST; FIB-4, Fibrosis index of 4 factors; LSM, liver-stiffness measurement; NPV, negative predictive value; PPV, positive predictive value; tAUC, time-dependent area under the operative characteristic curve.

#### Identification of low-risk NAFLD using FIB-4, LSM, and FAST score

The individual performance of FIB-4, LSM, and FAST score to identify low-risk NAFLD is summarized in Supplemental Table 8, http://links.lww.com/HC9/A486. In sequential testing, FIB-4 testing using a cutoff value of 1.3 first identified 59.4% of the patients as low-risk NAFLD, without missing any patients with LRE. In the second step, LSM with a cutoff value of 8 kPa identified 18.2% of the patients as low-risk NAFLD. In other words, sequential FIB-4 and LSM testing identified 77.6% of the cohort as low-risk NAFLD at the expense of missing out 3/27 (11.1%) LRE. Combining FIB-4 and LSM for all patients reduces the LRE to 0%, but the proportion of low-risk patients with NAFLD identified also reduced to 43.1%. FAST score identified a similar proportion of low-risk patients with NAFLD than the combination strategy (42.5%) at the expense of missing more LRE (6/27, 22.2%). These findings support a sequential approach of FIB-4 followed by LSM over the approach of using FIB-4 alone or using performing LSM for everyone.

## DISCUSSION

In this international study including 1262 patients with NAFLD followed up over a median of 3 years, we found that FIB-4 has excellent negative predictive value to predict LRE among patients with NAFLD, regardless of LSM. Further, no patients with low FIB-4 developed LREs or death, thus supporting the current guidelines of not performing VCTE among low-risk NAFLD patients even in the secondary or tertiary care setting. The performance of FIB-4 in predicting LRE and death was also similar to another European study involving 1173 patients with NAFLD.^19^

Most NAFLD guidelines recommend a sequential approach with FIB-4 followed by LSM in patients with intermediate or high FIB-4 because LSM has higher sensitivity and specificity for advanced fibrosis than FIB-4.^20^ However, it is unclear whether FIB-4 is a superior prognostic score than LSM, which is arguably the more clinically relevant question.^21^ Further, there are very limited data on how to interpret discordant results, such as high/intermediate FIB-4 with low LSM, or low FIB-4 with high LSM. Current guidelines recommend the use of liver biopsy in the setting of discordant results between FIB-4 and LSM.^5,22^ In practice, repeat LSM may be considered if there is concern over liver biopsy or unreliable LSM results due to elevated liver enzymes or high interquartile range. Here, we found that LSM does not outweigh FIB-4: patients with low FIB-4 have an extremely low risk of LREs regardless of LSM. Further, our findings highlight the disadvantages of VCTE in patients with low FIB-4: 89/910 (10%) of the patients with FIB-4 <1.3 had LSM > 12 kPa, yet none of these patients with LSM > 12 kPa had LREs during follow-up, suggesting that 10% of the patients with low FIB-4 are incorrectly identified as high risk based on LSM by VCTE. Similarly, the combination FIB-4/LSM approach demonstrated poorer risk stratification than the sequential approach (Supplemental Table S6, http://links.lww.com/HC9/A486).

Even in patients undergoing LSM by VCTE following FIB-4, we believe that LSM should not be considered the “superior” test, but rather LSM and FIB-4 should be considered complementary. We found that within each LSM category higher FIB-4 was associated with a dose-dependent increase in the incidence rate of LREs, and vice versa. Thus, combinations of NITs provide more prognostic information than individual NITs and FIB-4 has value even in patients who have undergone more specialized fibrosis assessment. The impact of FAST on LREs in patients with NAFLD has not to our knowledge been previously studied. FAST was designed as a noninvasive approach to identify patients with high-risk NASH (ie, NASH plus significant fibrosis) who may benefit from pharmacologic treatment, whereas FIB-4 and LSM were originally developed as noninvasive metrics of fibrosis stage, without accounting for “disease activity.”^11^ We found that FAST score was associated with LREs, but this association was relatively weak with lower tAUC than FIB-4 or LSM. These findings can be interpreted in two ways. First, we showed that FAST is measuring a clinically relevant parameter in that patients with higher FAST scores were more likely to develop LREs than those with low FAST. Second, consistent with prior literature on histologically defined NAFLD, steatohepatitis (as defined by FAST) is less predictive of adverse events than fibrosis stage (as defined by FIB-4 or LSM).^3^ Of note, our follow-up period was relatively short, while the effects of FAST-defined NASH may accumulate over time, FAST may have a greater impact after prolonged follow-up. In addition, whether patients with high FAST are more likely to respond to treatment than those with lower FAST scores is not known. Further studies will be required to understand the potential applications of the FAST score.

Our findings were contrary to an American study including 81,108 patients with NAFLD diagnosed using ICD code, which suggested that FIB-4 was an independent predictor of MACE.^23^ The difference in result is likely related to the younger age of patients with NAFLD (52 vs. 62 years) and lower rates of MACE (0.7% vs. 13.5%) in our cohort. Our findings were similar to the NASH Clinical Research Network cohort study, showing FIB-4 score was not associated with a higher incidence of MACE.^3^ Collectively, these findings suggest more data are needed before FIB-4 can be used to stratify MACE among NAFLD in a routine clinical setting.

Strengths of the study include the use of consecutive patients with NAFLD undergoing LSM in 2 countries and the use of hard clinical outcomes rather than surrogate measures of disease. We believe the diagnosis of NAFLD using radiological imaging is more accurate than using ICD code alone in other studies.^24,25^ All the clinical events were manually verified through chart review and validated with high accuracy. Limitations include that our cohorts were derived from secondary/tertiary care centers, though this limitation is intrinsic to nearly all real-world studies of LSM since VCTE is rarely done in a primary care setting. Due to the retrospective study design, we were unable to rule out excess alcohol intake not documented in the medical records or to fully assess baseline cardiac risk.

To conclude, FIB-4 has excellent negative predictive value to identify patients with NAFLD with low risk of LRE to be monitored in primary care setting. Our findings support the sequential approach of FIB-4 followed by LSM by VCTE recommended by most international guidelines and highlight the disadvantages of routine VCTE in patients with low FIB-4. In contrast, in higher-risk groups, the combination of FIB-4 and LSM can risk stratifying patients with NAFLD at risk of LRE beyond FIB-4 or LSM alone, with a high risk of LRE in patients with concordantly high FIB-4 and LSM.

## Supplementary Material

SUPPLEMENTARY MATERIAL
