# Supplementary material for: Combination of Fibrosis-4, liver-stiffness measurement, and Fibroscan-AST score to predict liver-related outcomes in nonalcoholic fatty liver disease
Source: Hepatol Commun. 2023 Sep 22;7(10):e0244. doi: 10.1097/HC9.0000000000000244 (PMC10519529; doi:10.1097/HC9.0000000000000244)
Supplement: SUPPLEMENTARY MATERIAL [file hc9-7-e0244-s001.docx]

**Supplementary Figure 1:** Study flowchart

**
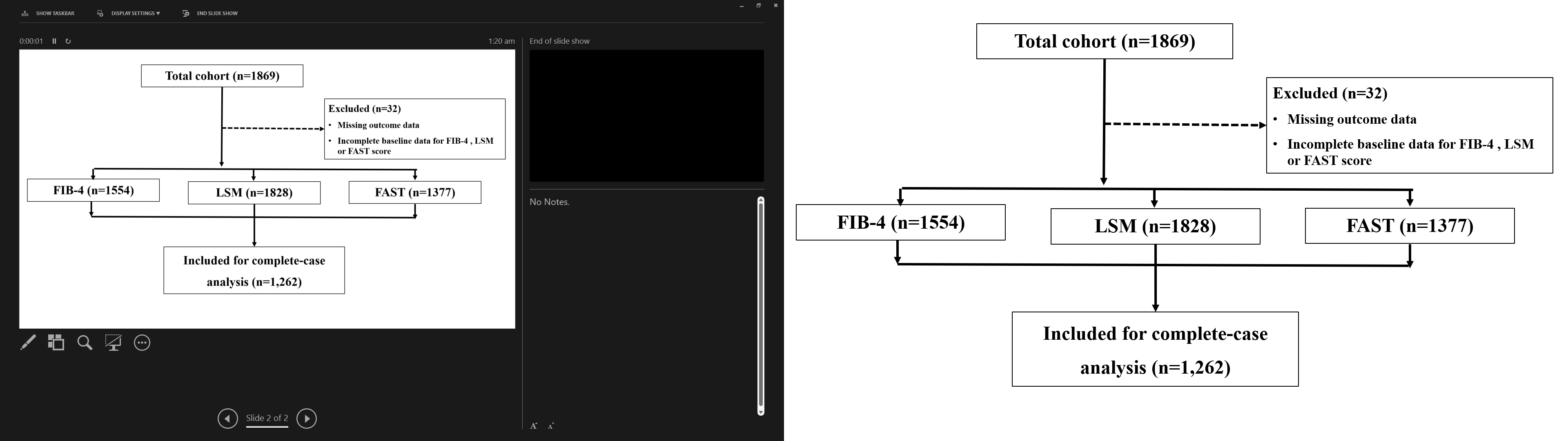
**

**Supplementary Figure 2:** Cumulative incidence of liver-related events or death, stratified by (a) FIB-4, (b) LSM and (c) FAST score
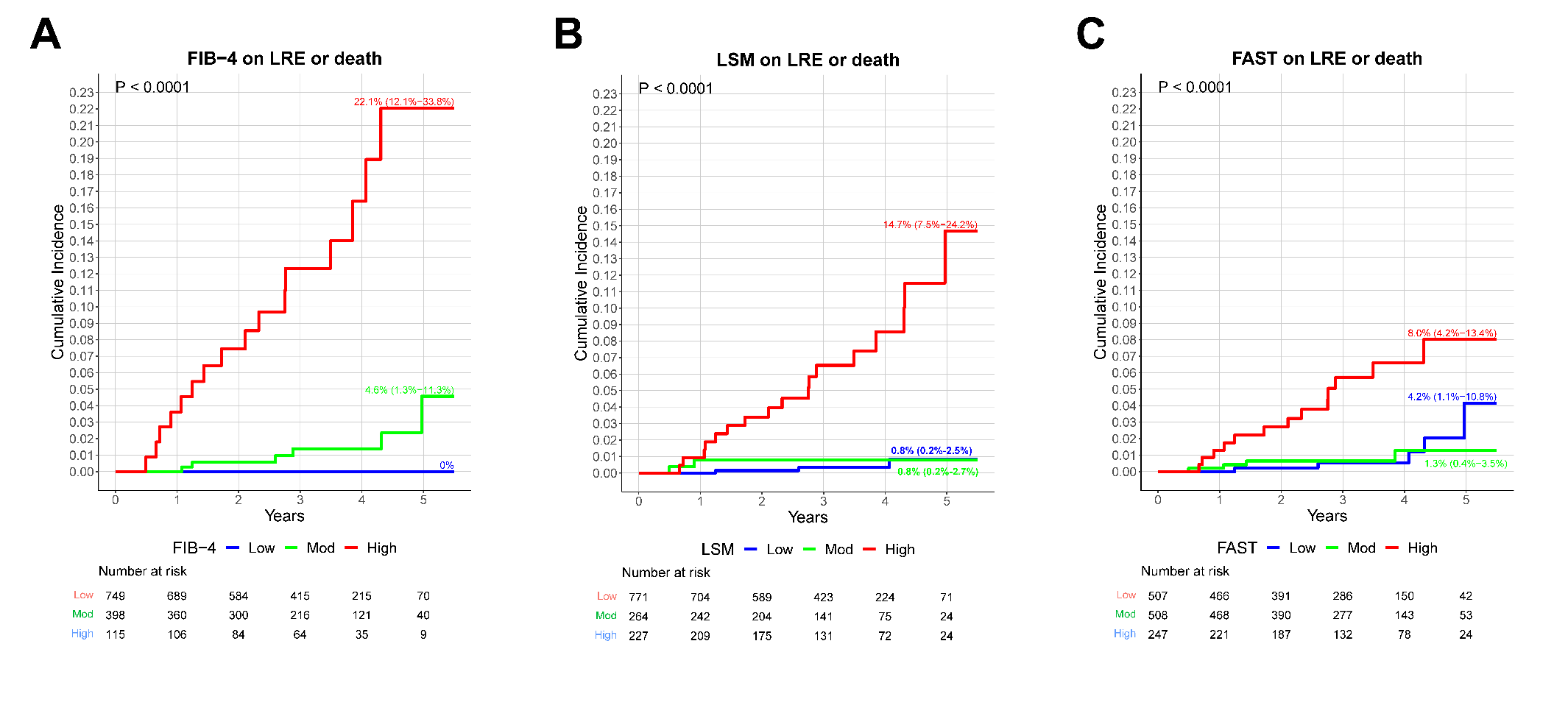


Abbreviations: LRE: liver related events, FIB-4: fibrosis index of 4 factor, LSM: liver stiffness measurement, FAST: FibroscanAST, Cumulative incidence of LRE were compared using competing risk analysis.

**Suplementary Table 1:** Number of liver-related events in NAFLD patients

| **Liver-related events** | **Total (n, %)** |
| --- | --- |
| Ascites | 21 (1.1) |
| Acute variceal bleeding | 9 (0.5) |
| Hepatic Encephalopathy | 10 (0.5) |
| Liver transplantation | 0 (0) |
| Hepatocellular carcinoma | 11 (0.6) |
| Total | 29 (1.6) |

*Note: some patients have more than one liver-related events

**Supplementary Table 2:** Multivariable analysis on the predictors for liver-related events

| **Predictors** | **Univariate analysis** | | **Multivariate analysis** | |
| --- | --- | --- | --- | --- |
|  | **OR (95%CI:)** | **p-value** | **OR (95% CI)** | **p-value** |
| Female | 1.2 (0.6-2.6) | 0.615 | 1.0 (0.4-2.5) | 0.919 |
| Obesity | 1.3 (0.5-2.9) | 0.603 | 1.2 (0.5-3.3) | 0.702 |
| Diabetes Mellitus | 4.1 (1.8-9.5) | 0.001 | 1.3 (0.5-3.4) | 0.648 |
| Hypertension | 7.5 (2.2-25.0) | 0.001 | 4.1 (1.1-15.3) | 0.033 |
| Bilirubin | 1.01 (0.998-1.02) | 0.103 | 1.0 (0.98-1.02) | 0.839 |
| Albumin | 0.87 (0.82-0.92) | < 0.001 | 0.93 (0.86-1.00) | 0.052 |
| FIB-4>2.67 | 23.5 (10.3-53.6) | < 0.001 | 9.8 (3.5-27.3) | < 0.001 |
| LSM>8kPa | 13.8 (4.1-46.1) | < 0.001 | 4.5 (1.1-17.6) | 0.031 |
| FAST>0.66 | 5.1 (2.4-11.1) | < 0.001 | 1.03 (0.4-2.9) | 0.963 |

Abbreviations: OR, odds ratio; CI, confidence interval, FIB-4, Fibrosis score-4; LSM, liver stiffness measurement, FAST, FibroscanAST

**Supplementary Table 3:** Causes of Death in NAFLD patients

| **Causes of death** | **Total (n, %)** |
| --- | --- |
| Cardiovascular disease | 4 (0.2) |
| Liver-related death | 3 (0.2) |
| Dementia | 1 (0.1) |
| Cancer | 1 (0.1) |
| Unknown | 2 (0.1) |
|  | 11 (0.6) |

**Supplementary Table 4:** The 5-year cumulative incidence of death and major cardiovascular adverse events stratified based on FIB-4, FAST score and liver-stiffness measurement

| **Death** | | **FIB-4** | | | | | | |  | |
| --- | --- | --- | --- | --- | --- | --- | --- | --- | --- | --- |
|  |  | **<1.3** | | **1.3-2.67** | | **>2.67** | |  | **All patients** | |
|  |  | 5-year cumulative incidence | Incidence rate per 1000 person years | 5-year cumulative incidence | Incidence rate per 1000 person years | 5-year cumulative incidence | Incidence rate per 1000 person years | p-value^#^ | 5-year cumulative incidence | Incidence rate per 1000 person years |
|  |  | (n = 910) |  | (n = 491) |  | (n = 145) |  |  |  |  |
| **LSM** | <8 kPa | 0% | 0  (0.0-0.3) | 1.4%  (0.3%-4.5%) | 3.18  (0.39-11.49) | 0% | 0.00  (0.00-51.73) | 0.054 | 0.4% (0.1%-1.2%) | 0.83  (0.10-3.01) |
|  | 8-12kPa | 0% | 0  (0.0-0.4) | 0% | 0.00  (0.00-11.34) | 16.3%  (4.8%-34.0%) | 47.12  (12.84-120.65) | < 0.001 | 1.8% (0.6%-4.3%) | 4.84  (1.32-12.39) |
|  | >12kPa | 0% | 0  (0.0-1.5) | 0% | 0.00  (0.00-12.48) | 0% | 0.00  (0.00-16.62) | NA | 0% | 0.00  (0.00-4.87) |
|  | p-value | NA | | 0.36 | | 0.0015 | | - | < 0.001 | |
|  | All patients | 0% | 0.00  (0.00-1.57) | 0.7%  (0.1%-2.3%) | 1.60  (0.19-5.78) | 3.9%  (1.2%-9.1%) | 10.58  (2.88-27.09) | <0.001 | 0.6%  (0.2%-1.2%) | 1.51  (0.55-3.28) |
| **FAST score** | < 0.35 | 0% | 0  (0.0-0.3) | 2.2%  (0.4%-7.2%) | 5.24  (0.63-18.91) | 0% | 0  (0.00-77.96) | 0.037 | 0.5%  (0.1%-1.8%) | 1.25  (0.15-4.52) |
|  | 0.35-0.67 | 0% | 0  (0.0-0.4) | 0% | 0  (0.00-6.99) | 7.6%  (1.2%-22.5%) | 19.70  (2.39-71.17) | < 0.001 | 0.5%  (0.1%-1.8%) | 1.26  (0.15-4.56) |
|  | >0.67 | 0% | 0  (0.0-1.5) | 0% | 0  (0.00-10.85) | 3.1%  (0.6%-9.7%) | 8.72  (1.06-31.51) | 0.073 | 0.9%  (0.2%-2.9%) | 2.50  (0.30-9.05) |
|  | p-value | NA | | 0.793 | | 0.436 | |  | 0.055 | |
|  | All patients | 0/749  (0%, 0.0-0.5) | 0  (0.0-0.14) | 4/398  (1.0%, 0.3-2.6) | 2.9  (1.1-7.8) | 4/115  (3.5%, 1.0-8.7) | 9.7  (3.7-25.9) | 0.386 | 8/1262  (0.6%, 0.3-1.2) | 1.8  (0.9-3.7) |
| **MACE** | | **FIB-4** | | | | | | |  | |
|  |  | <1.3 | | 1.3-2.67 | | >2.67 | |  | All patients | |
|  |  | 5-year cumulative incidence  (n = 693) | Incidence rate per 1000 person years | 5-year cumulative incidence  (n – 370) | Incidence rate per 1000 person years | 5-year cumulative incidence  (n = 115) | Incidence rate per 1000 person years | p-value^#^ | 5-year cumulative incidence | Incidence rate per 1000 person years |
| **LSM** | <8 kPa | 0.5% (0.1%-1.6%) | 1.19 (0.14-4.29) | 0.9% (0.1%-4.3% | 1.60 (0.04-8.93) | 0% | 0.00 (0.00-51.75) | 0.92 | 0.5% (0.2%-1.5%) | 1.26 (0.26-3.68) |
|  | 8-12kPa | 0% | 0.00 (0.00-8.90) | 4.6% (0.6%-15.4%) | 6.27 (0.76-22.66) | 0% | 0.00 (0.00-45.45) | 0.21 | 1.8% (0.3%-6.4%) | 2.46 (0.30-8.87) |
|  | >12kPa | 4.0% (0.7%-12.4%) | 8.61 (1.04-31.10) | 2.7% (0.5%-8.6%) | 6.82 (0.83-24.64) | 1.9% (0.1%-8.7%) | 4.55 (0.12-25.33) | 0.87 | 2.9% (1.1%-6.2%) | 6.71 (2.18-15.65) |
|  | p-value | 0.021 | | 0.40 | | 0.70 | | - | 0.037 | |
|  | All patients | 0.7% (0.2%-1.8%) | 1.72 (0.47-4.39) | 2.2% (0.7%-5.1%) | 4.05 (1.31-9.44) | 1.1% (0.1%-5.3%) | 2.69 (0.07-14.96) | 0.41 | 1.2% (0.6%-2.3%) | 2.54 (1.22-4.67) |
| **FAST** | < 0.35 | 0.7% (0.1%-2.2%) | 1.73 (0.21-6.24) | 1.4% (0.1%-6.7%) | 2.65 (0.07-14.75) | 0% | 0.00 (0.00-78.00) | 0.88 | 0.8% (0.2%-2.2%) | 1.89 (0.39-5.54) |
|  | 0.35-0.67 | 0% | 0.00 (0.00-3.89) | 3.3% (0.7%-9.4%) | 5.76 (1.19-16.84) | 0% | 0.00 (0.00-37.51) | 0.052 | 1.1% (0.2%-3.5%) | 1.91 (0.39-5.59) |
|  | >0.67 | 4.4% (0.8%-13.6%) | 8.94 (1.08-32.31) | 1.5% (0.1%-7.1%) | 2.97 (0.08-16.53) | 1.8% (0.1%-8.4%) | 4.41 (0.11-24.57) | 0.59 | 2.3% (0.8%-5.5%) | 5.08 (1.38-13.01) |
|  | p-value | 0.013 | | 0.73 | | 0.72 | |  | 0.28 | |
|  | All patients | 0.7% (0.2%-1.8%) | 1.72 (0.47-4.39) | 2.2% (0.7%-5.1%) | 4.05 (1.31-9.44) | 1.1% (0.1%-5.3%) | 0.7% (0.2%-1.8%) | 1.72 (0.47-4.39) | 2.2% (0.7%-5.1%) | 4.05 (1.31-9.44) |
| Abbreviations: FIB4: Fibrosis index 4, LSM: liver stiffness measurement, LRE: Liver-related events. | | | | | | | | | | |
| 5-year cumulative incidence is shown as events/number at risk (%, 95% CI). Incidence rate is shown as events in 1,000 per person-years (95% confidence interval) in the overall cohort.  Person-years was rounded to the nearest integer. Fisher-exact test for P-value of association for cumulative incidence between different subgroup of FIB-4 and LSM; NA – Not available  # comparing across incidence rate per 1000 person tears | | | | | | | | | | |

**Supplementary Table 5:** Accuracy of FIB-4, LSM and FAST score to predict clinical outcomes at 3 years with sensitivity ≥ 90%

|  | **tAUC^#^ (95% CI)** | **Cut-off** | **Sensitivity, % (95% CI)** | **Specificity, % (95% CI)** | **PPV, % (95% CI)** | **NPV, % (95% CI)** | **p-value for AUC comparison** |
| --- | --- | --- | --- | --- | --- | --- | --- |
| **Liver-related events** | | | | | | |  |
| FIB-4 | 0.775  (0.691-0.859) | 1.398 | 90.0  (68.3-98.8) | 65.0  (62.2-67.6) | 4.0  (2.4-6.2) | 99.8  (99.1-100) | ref |
| LSM | 0.778  (0.695-0.862) | 8.450 | 90.0  (68.3-98.8) | 65.6  (62.9-68.3) | 4.0  (2.4-6.3) | 99.8  (99.1-100) | 0.970 |
| FAST score | 0.579  (0.466-0.891) | 0.225 | 90.0  (68.3-98.8) | 25.8  (23.4-28.3) | 1.9  (1.1-3.0) | 99.4  (97.8-99.9) | 0.013 |
| **Liver-related events or death** | | | | | | |  |
| FIB-4 | 0.787  (0.721-0.854) | 1.397 | 92.6  (75.7-99.1) | 64.9  (62.1-67.5) | 5.4  (3.6-7.9) | 99.8  (99.1-100) | ref |
| LSM | 0.650  (0.565-0.734) | 5.750 | 92.6  (75.7-99.1) | 37.3  (34.6-40.1) | 3.1  (2.0-4.6) | 99.6  (98.4-99.9) | 0.010 |
| FAST score | 0.566  (0.467-0.664) | 0.195 | 92.6  (75.7-99.1) | 20.6  (18.3-22.9) | 2.5  (1.6-3.6) | 99.2  (97.2-99.9) | 0.014 |
| **Death** | | | | | | |  |
| FIB-4 | 0.801  (0.720-0.883) | 1.307 | 100  (63.1-100) | 60.3  (57.5-63.0) | 1.6  (0.7-3.1) | 100  (99.5-100) | ref |
| LSM | 0.523  (0.331-0.714) | 3.550 | 100  (63.1-100) | 4.5  (3.5-5.8) | 0.7  (0.3-1.3) | 100  (93.7) | 0.005 |
| FAST score | 0.584  (0.416-0.751) | 0.175 | 100  (63.1-100) | 16.7  (14.7-18.9) | 0.8  (0.3-1.5) | 100  (98.3-100) | 0.009 |
| **Major Adverse Cardiac Events** | | | | | | |  |
| FIB-4 | 0.501  (0.321-0.680) | 0.558 | 90.0  (55.5-99.7) | 10.1  (8.5-12.0) | 0.8  (0.4-1.5) | 99.2  (95.7-100) | ref |
| LSM | 0.538  (0.369-0.707) | 4.450 | 90.0  (55.5-99.7) | 17.6  (15.5-19.8) | 0.9  (0.4-1.6) | 99.5  (97.5-100) | 0.690 |
| FAST score | 0.500  (0.309-0.673) | 0.105 | 90.0  (55.5-99.7) | 8.2  (6.8-9.9) | 0.8  (0.3-1.5) | 99.0  (94.7-100) | 0.910 |
| *Abbreviations: ^#^* *tAUC, Time-dependent area under the received operating curve, PPV = positive predictive value, NPV = negative predictive value, CI = confidence interval. tAUC were compared using Delong test.* | | | | | | |  |

**Supplementary Table 6:** Accuracy of FIB-4, LSM and FAST score to predict clinical outcomes at 3 years with specificity ≥ 90%

|  | **tAUC^#^ (95% CI)** | **Cut-off** | **Sensitivity, % (95% CI)** | **Specificity, % (95% CI)** | **PPV, % (95% CI)** | **NPV, % (95% CI)** | **p-value for AUC comparison** |
| --- | --- | --- | --- | --- | --- | --- | --- |
| **Liver-related events** | | | | | | |  |
| FIB-4 | 0.825  (0.713-0.937) | 2.451 | 75.0  (50.1-91.3) | 90.0  (88.2-91.6) | 10.8  (6.2-17.2) | 99.6  (99.0-99.9) | ref |
| LSM | 0.700  (0.563-0.837 | 15.5 | 50.0  (27.2-72.8) | 90.0  (88.2-91.6) | 7.5  (3.6-13.3) | 99.1  (98.4-99.6) | 0.100 |
| FAST score | 0.675  (0.536-0.814) | 0.777 | 45.0  (23.1-68.5) | 90.0  (88.2-91.6) | 0.9  (3.1-12.5) | 99.0  (98.3-99.5) | 0.006 |
| **Liver-related events or death** | | | | | | |  |
| FIB-4 | 0.783  (0.677-0.889) | 2.435 | 66.7  (46.0-83.5) | 90.0  (88.1-91.6) | 12.7  (7.7-19.3) | 99.2  (98.5-99.6) | ref |
| LSM | 0.654  (0.534-0.774) | 15.5 | 40.7  (22.4-61.2) | 90.0  (88.2-91.7) | 8.2  (4.2-14.2) | 98.6  (97.7-99.2) | 0.020 |
| FAST score | 0.635  (0.515-0.716) | 0.777 | 37.0  (19.4-57.6) | 90.0  (88.2-91.7) | 7.5  (3.7-13.4) | 98.5  (97.6-99.1) | 0.010 |
| **Death** | | | | | | |  |
| FIB-4 | 0.700  (0.484-0.916) | 2.506 | 50.0  (15.7-84.3) | 90.0  (88.2-91.6) | 3.1  (0.8-7.7) | 99.6  (99.1-99.9) | ref |
| LSM | 0.500  (0.300-0.634) | 16.5 | 0.0  (0.0-36.9) | 90.4  (88.7-92.0) | 0.0  (0.0-3.0) | 99.3  (98.6-99.7) | 0.045 |
| FAST score | 0.513  (0.309-0.718) | 0.785 | 12.5  (0.3-52.7) | 90.2  (88.4-91.8) | 0.8  (0.02-4.4) | 99.4  (98.7-99.8) | 0.040 |
| **Major Adverse Cardiac Events** | | | | | | |  |
| FIB-4 | 0.500  (0.320-0.680) | 2.510 | 10.0  (0.3-44.5) | 90.0  (88.2-91.6) | 0.8  (0.02-4.3) | 99.2  (98.5-99.6) | ref |
| LSM | 0.650  (0.453-0.848) | 15.8 | 40.0  (12.1-73.8) | 90.1  (88.3-91.7) | 3.1  (0.9-7.8) | 99.5  (98.8-99.8) | 0.169 |
| FAST score | 0.551  (0.360-0.742) | 0.785 | 20.0  (2.5-55.6) | 90.3  (88.5-91.8) | 1.6  (0.2-5.7) | 99.3  (98.6-99.7) | 0.487 |
| *Abbreviations: ^#^ tAUC, Time-dependent area under the received operating curve, PPV = positive predictive value, NPV = negative predictive value, CI = confidence interval. tAUC were compared using Delong test.* | | | | | | |  |

**Supplementary Tabel 7**: Time-dependent ROC of FIB-4, LSM and FAST to predict clinical outcomes

|  | | **Liver-related events** | | | **Liver-related events or death** | | |
| --- | --- | --- | --- | --- | --- | --- | --- |
| Time (years) | NITs | tAUC (95% CI) | Pairwise comparison p value | | tAUC (95% CI) | Pairwise comparison | |
|  |  |  |  |  |  | p value | |
|  |  |  | LSM | FAST |  | LSM | FAST |
| 3 | FIB-4 | 0.902 (0.845-0.958) | 0.024 | 0.006 | 0.937 (0.881-0.994) | 0.143 | 0.034 |
|  | LSM | 0.774 (0.649-0.899) |  | 0.659 | 0.884 (0.812-0.956) |  | 0.279 |
|  | FAST | 0.757 (0.635-0.880) |  |  | 0.830 (0.702-0.958) |  |  |
| 5 | FIB-4 | 0.904 (0.857-0.950) | 0.026 | 0.001 | 0.925 (0.878-0.972) | 0.194 | 0.005 |
|  | LSM | 0.794 (0.694-0.895) |  | 0.161 | 0.871 (0.799-0.943) |  | 0.055 |
|  | FAST | 0.740 (0.631-0.848) |  |  | 0.778 (0.658-0.898) |  |  |

Abbreviations: *tAUC, Time-dependent Area under the received operating curve; NIT= non-invasive tests; FIB-4, Fibrosis index of 4-factors; LSM, liver stiffness measurement, FAST, FibroscanAST; CI = confidence interval; tAUC were compared using Delong test.*

**Supplementary Table 8:** Identification of low-risk NAFLD using FIB-4, LSM and FAST score

| **Non-invasive tests (NITs)** | **Cut-off of NITs** | **Low-risk NAFLD** | **Missed liver-related events or death** |
| --- | --- | --- | --- |
| FIB-4 alone | FIB-4 < 1.3,  LSM < 8kPa | 749/1262  (59.4%, 95%CI: 56.6-62.1) | 0/27  (0%, 95%CI: 0.0-12.8) |
| Sequential FIB-4 and LSM |  | 976/1262  (77.6%, 95%CI: 74.9-79.6) | 3/27  (11.1%, 95%CI: 2.3-29.2) |
| Combination FIB-4 and LSM |  | 544/1262  (43.1%, 95%CI: 40.4-45.9) | 0/27  (0%, 95%CI: 0.0-12.8) |
| FAST score | <0.35 | 536/1262  (42.5%, 95%CI: 39.7-45.3) | 6/27  (22.2%, 95%CI: 8.6-42.2) |

***Abbreviations:*** *NAFLD, non-alcoholic fatty liver disease; FIB-4, Fibrosis-index of 4 factors; LSM, liver-stiffness measurement, FAST, FibroscanAST*
